# Optimization of Vehicle Routing for Waste Collection and Transportation

**DOI:** 10.3390/ijerph17144963

**Published:** 2020-07-09

**Authors:** Hailin Wu, Fengming Tao, Bo Yang

**Affiliations:** 1 College of Mechanical Engineering, Chongqing University, Chongqing 400044, China; wu_hailin@foxmail.com; 2 School of Management Science and Real Estate, Chongqing University, Chongqing 400044, China; 3 College of Management, Chongqing Radio and Television University, Chongqing 400044, China; yangbo@cqdd.cq.cn

**Keywords:** waste collection and transportation, vehicle routing problem, waste bins with high priority, greenhouse gas emissions, waste filling level

## Abstract

For the sake of solving the optimization problem of urban waste collection and transportation in China, a priority considered green vehicle routing problem (PCGVRP) model in a waste management system is constructed in this paper, and specific algorithms are designed to solve the model. We pay particular concern to the possibility of immediate waste collection services for high-priority waste bins, e.g., those containing hospital or medical waste, because the harmful waste needs to be collected immediately. Otherwise, these may cause dangerous or negative effects. From the perspective of environmental protection, the proposed PCGVRP model considers both greenhouse gas (GHG) emission costs and conventional waste management costs. Waste filling level (WFL) is considered with the deployment of sensors on waste bins to realize dynamic routes instead of fixed routes, so that the economy and efficiency of waste collection and transportation can be improved. The optimal solution is obtained by a local search hybrid algorithm (LSHA), that is, the initial optimal solution is obtained by particle swarm optimization (PSO) and then a local search is performed on the initial optimal solution, which will be optimized by a simulated annealing (SA) algorithm by virtue of the global search capability. Several instances are selected from the database of capacitated vehicle routing problem (CVRP) so as to test and verify the effectiveness of the proposed LSHA algorithm. In addition, to obtain credible results and conclusions, a case using data about waste collection and transportation is employed to verify the PCGVRP model, and the effectiveness and practicability of the model was tested by setting a series of values of bins’ number with high priority and WFLs. The results show that (1) the proposed model can achieve a 42.3% reduction of negative effect compared with the traditional one; (2) a certain value of WFL between 60% and 80% can realize high efficiency of the waste collection and transportation; and (3) the best specific value of WFL is determined by the number of waste bins with high priority. Finally, some constructive propositions are put forward for the Environmental Protection Administration and waste management institutions based on these conclusions.

## 1. Introduction

Municipal solid waste (MSW) management is regarded as a challenging matter for contemporary cities [1,2] due to quick growth in the amount of waste, high waste collection costs [3], limited treatment capacities [4] and environmental problems [5]. In China, as the economy grows rapidly, the quantity of MSW has been growing significantly and the mean rate of growth has been around 3.5% [6]. There are several factors contributing to this social phenomenon such as urbanization [7], booming of population [7], and improvements of living standard [7], among others. Under such intricate circumstances, the application of operational research methods can make decision-makers benefit from programming [8].

MSW activities are grouped in five stages of waste life-cycle: generation, collection and transportation, transformation, treatment and final disposal [8,9]. The cost of waste collection and transportation accounts for 60–80% of total Waste Management System (WMS) costs, which is the critical factor in the fiscal spending of WMS, improvements in this field will play a significant role in saving municipal expenditure [10,11]. Thus, this article focuses on operational decisions at the second stage, collection and transportation. Waste collection refers to the use of waste collection vehicles to load waste from waste collection points. Waste transportation means the activity of taking the collected waste to the disposal center [12].

Moreover, some peculiar kinds of waste are supposed to be collected and transported with the least delay possible, because of their passive impact on people’s health, such as chemical waste, hospital waste, electronic waste (E-waste) [10] and waste close to gas stations and fuel stations [13] etc. In particular, during the global pandemic of COVID-19, the importance and necessity of giving priority to medical waste disposal are highlighted. Bins containing such waste should be collected as soon as possible to minimize the negative impact on the environment and human lives. In this regard, it is important to give high collection priority to such waste bins with negative effect, which will be taken into consideration as one of the critical factors in the proposed priority considered green vehicle routing problem (PCGVRP) model and solutions.

As a rule, the activity of waste collection and transportation is carried out by means of a fleet of vehicles aiming at emptying waste bins on the basis of predefined schedules [7,14,15]. However, this conventional waste collection is based on a lot of speculation about whether the filling levels of waste bins could vary from overflowing, partial filling, to completely emptying, which would lead to unnecessary resources consumption [16]. For these reasons, wireless sensor networks (WSN) have been deployed in MSW to achieve remote monitoring filling levels of waste bins [17]. In the meantime, waste collection trucks can communicate with waste bins with sensors by the Internet of things system to acquire the data about the status of bins [18,19,20].

Lastly, it is worthy of mention that the growth of waste is closely related to environmental deterioration [7], which is because that the activity of waste collection and transportation consumes a lot of fuel resulting in GHG emissions [7,21,22,23].Therefore, taking into account its effect on the environment, sustainable management of waste collection and transportation with the objective of minimizing GHG emissions is indispensable both for resource savings and environmental conservation [24].

Due to the increasing amount of waste and the increasing difficulty of SWM, many areas have established laws on waste collection and transportation, including all kinds of waste such as hazardous waste [25], chemical waste [26], E-waste [27], roundwood waste [28], construction waste [29], and so on. In particular, there has been an increase in laws and regulations on hazardous waste and E-waste collection [30,31] over the past two decades, for their particularity and harmfulness. Generally speaking, the purpose of this legislation is to reduce its impact on the environment. Therefore, we can see that waste collection and transportation has been paid increasing attention in legislation and by citizens.

This paper concentrates on collecting waste from waste bins and transporting them to waste disposal centers. More specifically, we propose a waste collection and transportation model that gives high collection priority to specific waste. We replace conventional fixed routes with dynamic systems that respond to the actual filling levels of sensor-based waste bins. This allows us to reduce the probability of collecting overflowing or empty waste bins. In the meantime, we consider the reduction of GHG emissions in the model and, accordingly, design a better and greener PCGVRP model. To sum up, this paper aims to reduce the cost, GHG emissions and negative impact of waste in the process of waste collection and transportation. The novelty lies in the consideration of the collection priority of different waste and the application of sensor-based waste bins.

## 2. Literature Review

### 2.1. Research about Waste Collection and Transportation 

A vehicle routing problem (VRP) is about the route optimization problem introduced by Dantzig and Ramser [32] and was applied in the field of waste collection and transportation by Beltrami and Bodin [33]. Vu et al. [34] studied route optimization of waste collection and transportation and found that travel distance was the main research factor. Rızvanoğlu et al., used linear programming and geographic information system analysis to determine the beat routes of waste collection and transportation and concluded with better linear programming. Yadava and Karmakarb [35] proposed plausible mathematical and computational modeling approached for sustainable collection and transportation of municipal solid waste. Miranda et al. [36] developed and implied a mixed integer linear optimization model for a waste collection system for serving a rural archipelago. Zhao et al. [37] studied the location and routes problem for hazardous waste with the objective of minimizing cost and risk.

The traditional method of waste collection and transportation refers to taking all the waste bins and transport waste to the disposal station by trucks along the settled routes [38]. This process involves labor costs, fuel costs, maintenance costs, etc., so the cost is very high, accounting for most of the SWM spending [38]. For these reasons, a lot of research proposed an approach to collect waste according to the filling levels of waste bins, which are predicted based on either historical data [7,17,39] or sensory data [18,38] obtained from waste bins and trucks [38]. Abdallah et al. [38] developed a selection procedure for waste bins to be collected, which have high filling levels based on historical data. Mamun et al. [17] presented a waste bin monitoring system, which is supported by a sensor technology and interaction system and the experimental results showed that the system can assist in an optimization model for route optimization.

Taking into account the impact on the environment of logistics, waste collection and transportation has gained greater attention in recent years. Leggieria and Haouari [40] established a model of Green VRP considering environmental issues and exact approach was proposed to solve it. Herdari et. al [41] took GHG emissions of vehicles into account which was calculated by the flow of WSM and emission coefficient. Mohsenizadeh et al. [24] developed a bi-objective model to investigate the impact of $CO_{2}$ emissions from transportation activities of MSW. Reddy et al. [42] proposed a model to decide facility locations and vehicle routes while accounting for carbon footprint.

It can be seen from the above that there is a wealth of research about waste collection and transportation and some design routes according to filling levels of waste bins from historical data or sensors with environmental concerns. However, the impact of different filling levels of sensor-based bins including costs, collected waste percentage and so on has been rarely considered especially when taking waste priority into account. Furthermore, it is important to plan the optimal route for high-priority waste because of its negative effect, which has been rarely studied.

### 2.2. Research about Priority in a Vehicle Routing Problem (VRP)

Nesmachnow et al. [8] built a waste collection model considering priorities with two objectives, the shortest distance and the best service respectively. Anagnostopoulos et al. [13] developed and compared four models for waste collection and transportation considering waste bins with high priority and found that different models are applicable to different situations. Tirkolaee et al. [10] built a model of waste collection and transportation, and gave the collection priority, which was realized by time windows, to some designated sites, including medical centers, hospitals and chemical plants that might generate harmful waste that needs to be collected as soon as possible. Armas et al. [43] built a rich VRP model taking the customer priority into account for a trucking enterprise in Spain and which is solved by a heuristic algorithm. Molina et al. [44] proposed a comprehensive model with the definition of different service priorities so as to cope with orders of high priorities as much as possible. Wang et al. [45] integrated customer service priority into the dynamic programming approach to optimize vehicle routes by order preference by similarity to ideal solution (TOPSIS).

From the above studies, we can see priority has been considered in some areas, including customer service, waste management and so on. Therefore, it is necessary to give thought to priority in the process of waste collection and transportation. However, few articles take consideration of the negative effects of delayed waste collection.

### 2.3. Research about Algorithms

VRP has been extensively and deeply studied ever since the 1960s, and a series of solving methods have emerged, including the exact method, heuristic method and meta-heuristic method [43]. Owing to the complexity of VRPs, it is not efficient to solve it with exact methods [46]. Hence, the research on heuristic method and meta-heuristic method is increasingly rich [43].

Along with the discrete particle swarm optimization (PSO), Rau et al. [47] developed a heuristic method to improve the solution quality of PSO particle to solve a multi-objective problem. Tirkolaee et al. [10] solved a vehicle routing problem with time window (VRPTW) of waste collection by a simulated annealing algorithm. Tirkolaee et al. [11] designed an improved ant colony algorithm for the proposed model of a capacitated arc routing problem (CARP). Wichapa and Khokhajaikia [48] designed hybrid genetic algorithm (HGA) to solve VRP for infectious waste transportation. Delgado-Antequera et al. [34] proposed an integrated greedy algorithm coupled with a variable neighborhood search for a multi-objective routing problem for waste collection and transportation.

To sum up, there are lots of research of algorithms to solve the waste collection problem. Nevertheless, they are mostly single algorithms instead of hybrid algorithms that can make the best of both worlds. The algorithm involved in this paper combines the high efficiency of PSO and the global optimization capability of simulated annealing (SA) performing better than a single algorithm. With the consideration of the main characteristics and gaps of the research literature, the paper proposed an integrated model considering priority and GHG emissions based on waste filling-level data from sensors for waste collection and transportation, and a hybrid algorithm is designed to solve the model.

## 3. Model Formulation

### 3.1. Problem Description

The problem involves obtaining the optimal paths of each vehicle with the objective of minimizing the total distance, total GHG emissions, total comprehensive costs including vehicle costs and GHG emissions costs. Waste bins located in specific areas (e.g., hospital, fuel station, gas station) are characterized as high priority bins which should be collected as soon as possible. The vehicles are located at the disposal center and start their trips toward the allocated waste bins. When the waste collection vehicle is fully loaded or the collection task is completed, it must get back to the disposal center so as to upload the collected waste.

Apart from the above description we make following assumptions:

- Each waste bin is only collected by one vehicle once.
- There is one disposal center.
- The vehicles must depart from the disposal center and go back to the disposal center when the task ends.
- There is single type of waste collection vehicle.
- The location of the disposal center and each waste bin are known.

The problem formulation considers the following elements:

- Each bin, $B=\left\{b_{1},b_{2},\cdots b_{n}\right\}$, has a collection priority, which is determined in accordance with the passive influence of waste.
- A set of vehicles $V=\left\{v_{1},\;v_{2}\;\cdots v_{m}\right\}$ to collect waste, with a maximum capacity.
- A disposal center D where vehicles star and end their trips.
- A set of waste bin filling level $L=\left\{l_{1},\;l_{2}\cdots l_{n}\right\}$, for $\forall \;i\in \left\{1,2,\;\cdots ,n\right\},\;l_{i}\in \left[0,1\right]$, where $l_{i}$ indicates the percentage of waste bin $b_{i}$ filled by waste.

### 3.2. Notation

The notations and descriptions are shown in Table 1.

### 3.3. Analysis of Objective Function

The PCGVRP model of waste collection and transportation in this paper considers three kinds of objectives: minimize total distance (T_D), minimize total GHG emissions ($T\_E_{GHG}$) and total costs (T_C) including vehicles costs and GHG emissions costs in this paper. Firstly, we analyze the three objective functions respectively and describe them as mathematical expressions. On this basis, the PCGVRP model is further determined by the analysis.

#### 3.3.1. Total Distance

(1) $$T\_D=\underset{i\in \left(B\cup^{}D\right)}{\sum }\underset{j\in \left(B\cup^{}D\right)}{\sum }x_{ij}^{k}d_{ij}$$

#### 3.3.2. Total Greenhouse Gas (GHG) Emissions

GHG is the emission from fossil fuel consumption in the process of waste collection and transportation [12] and usually the environmental effect of GHG emissions is approximated by $CO_{2}$ equivalents ($CO_{2}e$). Furthermore, GHG emissions show an approximately linear relation to the fuel consumption of a vehicle [22], we estimate GHG emissions based on fuel consumption and express its effect in terms of $CO_{2}$ during waste collection and transportation activities.

According to the analysis of literature [22], considering the linear relationship of load and GHG emissions, the GHG emissions can be expressed as follows:

(2) $$E_{GHG}\left(Q\right)=e*d*r\left(Q\right)$$

The fuel consumption rate (FCR) of the vehicle is linearly related to the vehicle load (Q), which can be expressed by the below equation [49]:

(3) $$r\left(Q\right)=r_{0}+(\left(r^{*}-r_{0})/C_{p}\right)*Q$$

Therefore, the FCR when vehicle goes from waste bin $b_{i}$ to $b_{j}$ can be expressed as:

(4) $$r_{ij}=r_{0}+(\left(r^{*}-r_{0})/C_{p}\right)*\left(\underset{i\in \left(B\cup^{}D\right)}{\sum }\underset{j\in \left(B\cup^{}D\right)}{\sum }x_{ij}^{k}q_{j}\right)$$

In the PCGVRP model, the total GHG emissions can be expressed as:

(5) $$E_{GHG}=e\underset{i\in B}{\sum }\underset{j\in B}{\sum }(d_{ij}*r_{0}+(\left(r^{*}-r_{0})/C_{p}\right)*\left(\underset{i\in \left(B\cup^{}D\right)}{\sum }\underset{j\in \left(B\cup^{}D\right)}{\sum }x_{ij}^{k}q_{j}\right))$$

#### 3.3.3. Total Costs

The objectives of minimizing T_D and $T\_E_{GHG}$ can only optimize routes either from the environmental point or from the economic point. However, the objective of T_C can take the both into consideration, which includes vehicle costs and GHG emissions costs. Vehicle costs can be divided into fixed vehicle costs and variable vehicle costs. Fixed costs mean the relatively fixed cost in the working process of waste collection vehicles, such as depreciation expenses, all taxes and fees, driver’s salary and so on, which can be calculated as:

(6) $$C_{fixed}=\underset{k\in V}{\sum }\underset{j\in \left(B\cup^{}D\right)}{\sum }x_{0j}^{k}P_{fixed}$$

Variable costs refer to the cost of fuel from driving between the collection nodes.

(7) $$C_{fuel}=\underset{i\in \left(B\cup^{}D\right)}{\sum }\underset{j\in \left(B\cup^{}D\right)}{\sum }x_{ij}^{k}d_{ij}r_{ij}P_{fuel}$$

Therefore, vehicle costs can be expressed as follows:

(8) $$C_{vehicle}=C_{fixed}+C_{fuel}=\underset{k\in V}{\sum }\underset{j\in \left(B\cup^{}D\right)}{\sum }x_{0j}^{k}P_{fixed}+\underset{i\in \left(B\cup^{}D\right)}{\sum }\underset{j\in \left(B\cup^{}D\right)}{\sum }x_{ij}^{k}d_{ij}r_{ij}P_{fuel}$$

GHG emissions can be translated into GHG emissions costs by carbon price. Thus, GHG emissions costs can be calculated by:

(9) $$C_{GHG}=\epsilon E_{GHG}=\epsilon e\underset{i\in B}{\sum }\underset{j\in B}{\sum }(d_{ij}*r_{0}+(\left(r^{*}-r_{0})/C_{p}\right)*\left(\underset{i\in \left(B\cup^{}D\right)}{\sum }\underset{j\in \left(B\cup^{}D\right)}{\sum }x_{ij}^{k}q_{j}\right))$$

Total costs can be calculated as:

(10) $$T\_C=C_{vehicle}+C_{GHG}$$

### 3.4. Model Construction

In accordance with the above analysis, the mathematical expressions of the PCGVRP model are as below:

(11) $$Min\;T\_D=\underset{i\in \left(B\cup^{}D\right)}{\sum }\underset{j\in \left(B\cup^{}D\right)}{\sum }x_{ij}^{k}d_{ij}$$

(12) $$Min\;T\_E_{GHG}=e\underset{i\in B}{\sum }\underset{j\in B}{\sum }(d_{ij}*r_{0}+(\left(r^{*}-r_{0})/C_{p}\right)*\left(\underset{i\in \left(B\cup^{}D\right)}{\sum }\underset{j\in \left(B\cup^{}D\right)}{\sum }x_{ij}^{k}q_{j}\right))$$

(13) $$Min\;T\_C=\underset{k\in V}{\sum }\underset{j\in \left(B\cup^{}D\right)}{\sum }x_{0j}^{k}P_{fixed}+\underset{i\in \left(B\cup^{}D\right)}{\sum }\underset{j\in \left(B\cup^{}D\right)}{\sum }x_{ij}^{k}d_{ij}r_{ij}P_{fuel}+\;\epsilon e\underset{i\in B}{\sum }\underset{j\in B}{\sum }(d_{ij}*r_{0}+(\left(r^{*}-r_{0})/C_{p}\right)*\left(\underset{i\in \left(B\cup^{}D\right)}{\sum }\underset{j\in \left(B\cup^{}D\right)}{\sum }x_{ij}^{k}q_{j}\right))$$

Subject to:

(14) $$\underset{k\in V}{\sum }\underset{i\in \left(B\cup^{}D\right)}{\sum }x_{ij}^{k}=1,\forall j\in \left(B\cup^{}D\right)$$

(15) $$\underset{k\in V}{\sum }\underset{j\in \left(B\cup^{}D\right)}{\sum }x_{ij}^{k}=1,\forall i\in \left(B\cup^{}D\right)$$

(16) $$\underset{i\in \left(B\cup^{}D\right)}{\sum }x_{ij}^{k}=\underset{j\in \left(B\cup^{}D\right)}{\sum }x_{ij}^{k}=1,\forall i\in \left(B\cup^{}D\right),k\in V$$

(17) $$\underset{i\in \left(B\cup^{}D\right)}{\sum }\underset{j\in \left(B\cup^{}D\right)}{\sum }x_{ij}^{k}q_{j}\leq C_{p},\forall \;k\in V$$

(18) $$(\lambda_{i}-\lambda_{j})\left(t_{i}^{k}-t_{j}^{k}\right)\leq 0,\forall i,j\in B,\;k\in V$$

(19) $$\underset{i\in \left(B\cup^{}D\right)}{\sum }\underset{j\in \left(B\cup^{}D\right)}{\sum }x_{ij}^{k}\leq \left|S\right|-1,S⊑\left\{1,2,\cdots ,N\right\},\;S\neq \left\{\;\right\},\forall \;k\in V$$

(20) $$x_{ij}^{k}\in \left\{0,1\right\},\forall i,j\in \left(B\cup^{}D\right),\;k\in V$$

Firstly, the three objective functions (11)–(13) are to minimize total distance, total GHG emissions and total costs, respectively, whereas each waste bin is only collected once by one vehicle, as stated by constraint (14). A vehicle starts from the disposal center and goes back to the disposal center after visiting a waste bin, which is imposed by Equations (15)–(17) guarantees that the maximum capacity is respected by all routes. Constraint (18) ensures the collection service for the high priority waste bins while Constraint (19) eliminates sub-tours. Finally, Equation (20) specifies the types of the variables.

## 4. Algorithm

### 4.1. Algorithm Design

**Figure 1:**
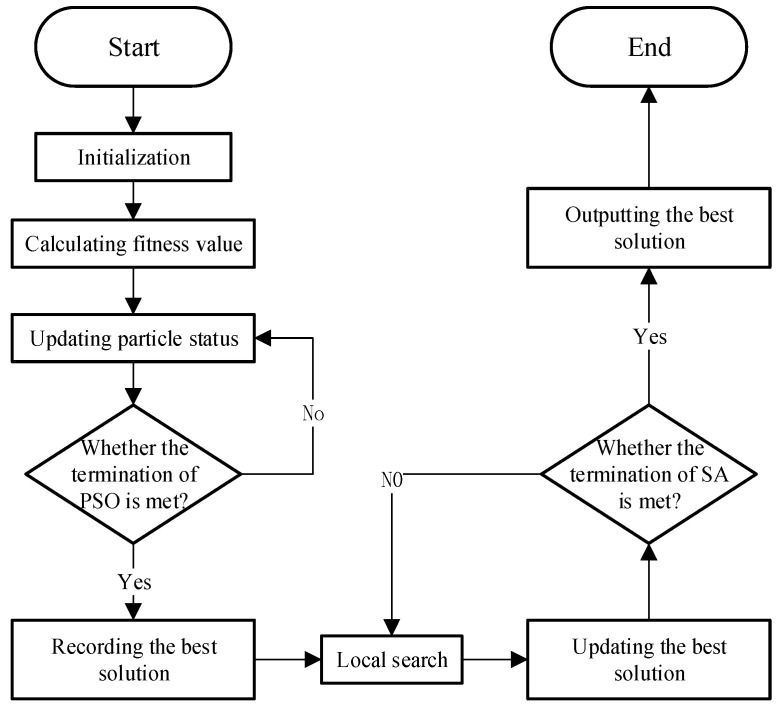
Flowchart of local search hybrid algorithm (LSHA).

The exact solution method is inefficient for solving the medium and large VRPs in real life [43]. For this reason, we pay attention to meta-heuristic methods which can generate suitable high-quality solutions within a rational computational time. In order to obtain high-quality solutions to practical problems, this paper proposes a hybrid local search algorithm based on PSO and SA algorithms. Its basic process is shown in Figure 1.

Firstly, the PSO algorithm is applied to generate an initial solution, and then local search will be operated to produce new solutions based on the initial solution. Finally, a SA with the ability to escape from local optimums is deployed to decide the optimal global solution.

### 4.2. Particle Coding and Decoding

For a vehicle routing problem with n waste bins, a 2n dimension space is constructed, and each waste bin corresponds to a two-dimensional value: (1) the vehicle number a that completes the waste bin collection; (2) the order b of the waste bin in the route of vehicle a. That is, (1) the position P of each particle is a 2n dimension vector: where $X_{a}$ represents the collection vehicle corresponding to each bin in the waste collection service, total n dimensions; (2) $X_{b}$ represents the order of each waste bin in the corresponding vehicle route, total n dimension. For example, suppose the number of waste bins to be collected in a waste collection activity is 10, and there are three vehicles in charge of waste collection. If, at a certain time, the position vector of a particle is shown in Table 2.

Taking waste bin 3 as an example, $X_{a}$ dimension is 1, which means that the waste bin 3 is collected by the vehicle 1; $X_{b}$ dimension is 2, which means that the order of waste bin 3 in the route of vehicle 1 is 2, and so on; the corresponding solution of this particle is shown in Table 3. 

### 4.3. Constructing Initial Optimal Solution Based on Particle Swarm Optimization (PSO) Algorithm

#### 4.3.1. Initialization and Fitness Function

- Initialization

Set the parameter of PSO as shown in Table 4, and the initialized position and velocity of the ith population is described as Equation (21).

(21) $$x_{i}=rand\left(lPar\right).*\left(x_{max}-x_{min}\right)+x_{min}\;v_{i}=rand\left(lPar\right).*\left(v_{max}-v_{min}\right)+x_{min}$$

- Fitness Function

The fitness function value is a quantitative index for judging the pros and cons of the particle position. According to the three kinds of objective functions in Section 3.3, the three corresponding types of fitness functions can be constructed as Equations (22)–(24).

(22) $$\begin{array}{ll} {Fitness}_{D}=T_{\_}D & +M\underset{k\in V}{\sum }\max\left(\underset{i\in \left(B\cup^{}D\right)}{\sum }\underset{j\in \left(B\cup^{}D\right)}{\sum }x_{ij}^{k}q_{j}-C_{p},0\right)\\ & +M\underset{k\in V}{\sum }\max\left(\left(\lambda_{i}-\lambda_{j}\right)\left(t_{i}^{k}-t_{j}^{k}\right),0\right) \end{array}$$

(23) $$\begin{array}{ll} {Fitness}_{E}=T_{\_}E & +M\underset{k\in V}{\sum }\max\left(\underset{i\in \left(B\cup^{}D\right)}{\sum }\underset{j\in \left(B\cup^{}D\right)}{\sum }x_{ij}^{k}q_{j}-C_{p},0\right)\\ & +M\underset{k\in V}{\sum }\max\left(\left(\lambda_{i}-\lambda_{j}\right)\left(t_{i}^{k}-t_{j}^{k}\right),0\right) \end{array}$$

(24) $$\begin{array}{ll} {Fitness}_{C}=T_{\_}C & +M\underset{k\in V}{\sum }\max\left(\underset{i\in \left(B\cup^{}D\right)}{\sum }\underset{j\in \left(B\cup^{}D\right)}{\sum }x_{ij}^{k}q_{j}-C_{p},0\right)\\ & +M\underset{k\in V}{\sum }\max\left(\left(\lambda_{i}-\lambda_{j}\right)\left(t_{i}^{k}-t_{j}^{k}\right),0\right) \end{array}$$

Each fitness function can be divided into three parts:

The first part is the objective function: total distance $T_{\_}D$ in Equation (22); total GHG emissions $T_{\_}E$ in Equation (23); total costs $T_{\_}C$ in Equation (24).

The second part, $M\sum_{k\in V}\max\left(\sum_{i\in \left(B\cup^{}D\right)}\sum_{j\in \left(B\cup^{}D\right)}x_{ij}^{k}q_{j}-C_{p},0\right)$ is the treatment of vehicle load constraints where M is a very large positive number. When the solution corresponding to the position of a certain particle is overloaded as an infeasible solution, that is, $\sum_{i\in \left(B\cup^{}D\right)}\sum_{j\in \left(B\cup^{}D\right)}x_{ij}^{k}q_{j}>0,M$ can make the overall fitness value of the particle larger, so that the solution corresponding to this position is eliminated. In this way, we can avoid the situation where an infeasible solution can survive due to overload.

The third part, $M\sum_{k\in V}\max\left(\left(\lambda_{i}-\lambda_{j}\right)\left(t_{i}^{k}-t_{j}^{k}\right),0\right)$ is the priority treatment of specific waste bins. When the solution does not give the priority the specific waste bins, that is, $\left(\lambda_{i}-\lambda_{j}\right)\left(t_{i}^{k}-t_{j}^{k}\right)>0$, M can make the overall fitness value of the particle larger, so that the solution corresponding to this position is eliminated. In this way, we can promise priority to the specific waste bins.

#### 4.3.2. Obtaining Optimal Solution

In each iteration, each particle records its current optimal value through comparison, indicated as $p_{i}^{best}\left(t\right)$, and all particles have a common global optimal value, indicated as $g^{best}\left(t\right)$.

#### 4.3.3. Particle Status Update

For each particle, update velocity according to Equation (25), and when velocity exceeds the range, take the value according to the boundary as shown by Equation (26).

(25) $$v_{i}\left(t+1\right)=\omega_{i}\left(t\right)+c_{1}r_{1}\left(p_{i}^{best}\left(t\right)-x_{i}\left(t\right)\right)+c_{2}r_{2}\left(g^{best}\left(t\right)-x_{i}\left(t\right)\right)$$

(26) $$\{\begin{array}{c} if\;v_{i}\left(t+1\right)>v_{max},\;v_{i}\left(t+1\right)=v_{max}\\ if\;v_{i}\left(t+1\right)<v_{max},\;v_{i}\left(t+1\right)=v_{min} \end{array}$$

After updating velocity, the position of particle i will be updated according to Equation (27).

(27) $$x_{i}\left(t+1\right)=x_{i}\left(t\right)+v_{i}\left(t+1\right)$$

In Equation (25), inertia weight (ω) is the ability of the particle to remain in motion at the previous moment and is very important in PSO algorithm. In this paper, a time-varying weight is used which is expressed in Equation (28).

(28) $$\omega \left(t+1\right)=\omega \left(t\right)*r_{\omega }$$

#### 4.3.4. Terminating Condition

Finally, the appearing of population quantity nPop is the termination of the evolutionary.

### 4.4. Local Search

**Figure 2:**
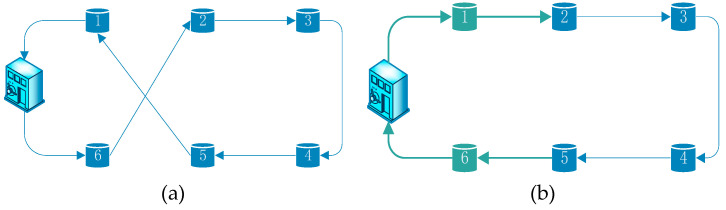
Swap operation. (**a**) Route before swap operation and (**b**) route after swap operation.

Next, a series of local search operations will be performed on the optimal individuals generated by PSO algorithm so as to improve the quality of the final optimal solution. Three local search operations designed in this paper are as below:

Swap operation: if a random number p∈(0,1/3], two points will be chosen at random in the present coding sequence. Next the positions of two selected points are exchange. As we can see in Figure 2, if the two selected points are 1 and 6, “623451” will be exchange for “123456”.

**Figure 3:**
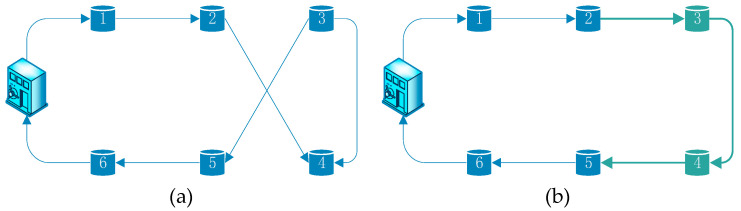
Reverse operation. (**a**) Route before reverse operation and (**b**) route after reverse operation.

Reverse operation: if a random number p∈(1/3,2/3], two points will be chosen at random in the present coding sequence. Next, the point sequence between the two selected points is reversed. As we can see in Figure 3, if the two point are 2 and 5, “2435” will be exchange for “2345”.

**Figure 4:**
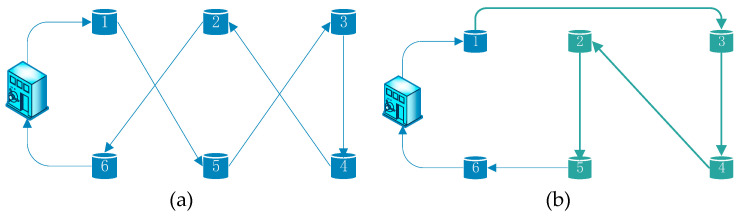
Insert operation. (**a**) Route before insert operation and (**b**) route after insert operation.

Insert operation: if a random number p∈(2/3,1], two points will be chosen at random in the present coding sequence, and then the first selected point will be inserted after the second selected point. As we can see in Figure 4, if the two point are 5 and 2, “5342” will be exchanged for “3425”.

### 4.5. Obtain Optimal Solution Using Simulated Annealing (SA) Algorithm

Fort the new solutions obtained by local search operations, the SA algorithm is considered to decide the optimal solution. In this section, the Metropolis criterion is applied to new solutions and obtained local search operations, to decide whether to accept the new solution, as shown in Equation (29). p represents the possibility to accept new solution. When ∆f>0, that is new solution is worse than the original solution, the possibility to accept it is $\exp\left(-∆f/T_{i}\right)$; When ∆f≤0, that is new solution is better than the original solution, the possibility to accept it is 1. When the temperature reaches the final temperature, the algorithm ends.

(29) $$p=\{\begin{array}{c} \exp\left(-∆f/T_{i}\right),∆f>0\\ 1,∆f\leq 0 \end{array}$$

## 5. Numerical Experiment

The calculation results in this part are all executed by a notebook computer equipped with an Intel core i5−8250U @1.60GHz and 8 GB of RAM. The solution algorithm is developed 20 times and the best solution is used.

### 5.1. Algorithm Experiment

#### 5.1.1. Test Cases

Here, the capacitated vehicle routing problem (CVRP) benchmark database (Dataset: Christofides and Eilon, 1969) is employed to test and verify the effectiveness of LSHA. This paper randomly selects 8 cases from the database including small-scale study (Pro1, Pro2, Pro3, Pro4), medium-scale study (Pro5, Pro6, Pro7) and large-scale study (Pro8), the detailed information is shown in Table 5.

#### 5.1.2. Parameters Setting

Parameters of vehicles are shown in Table 6 according to references [21,50,51] and parameters of the proposed algorithm are shown in Table 7 according to references [10,52,53,54].

#### 5.1.3. Results of Algorithm Experiment

**Figure 5:**
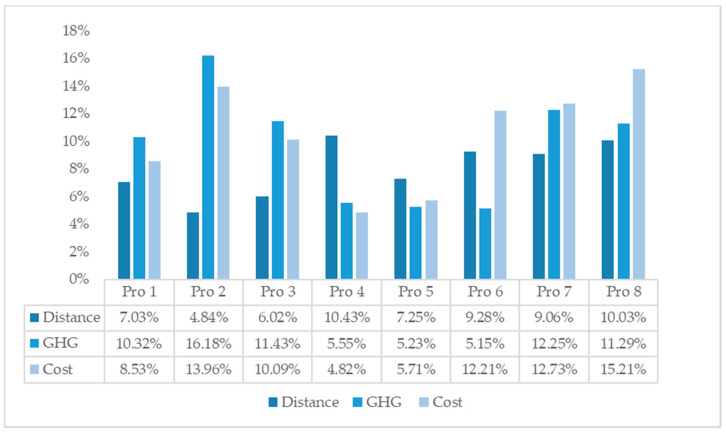
Savings of distance, GHG emissions and cost.

The results of PSO and the proposed algorithm are compared in Table 8, including total distance, total GHG emissions and total costs. To be clear, Figure 5 gives the distance saving, GHG emissions saving and costs saving of the proposed algorithm LSHA compared with PSO. We can see from Table 6 and Figure 5 that the proposed algorithm outperforms in all three areas. 

### 5.2. Model Experiment

#### 5.2.1. Experimental Design

**Figure 6:**
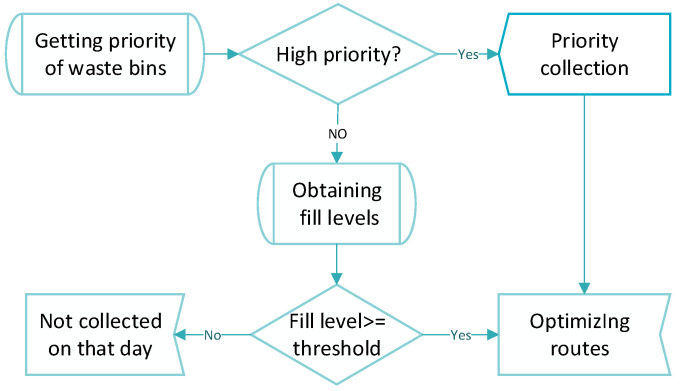
Flowchart of priority and knowledge-based decision making.

This paper is concentrated on the optimization and simulation of waste collection and transportation considering waste bins’ priority. The proposed PCGVRP model is developed in line with the different priorities of waste bins and real-time waste-filling levels of sensor-based waste bins. The contents of route optimizations of waste collection and transportation are as follows: (1) guaranteeing priority collection of specific waste bins; (2) reducing the number of waste bins collected in one trip based on the actual filling levels of general waste bins; (3) minimizing the distance, GHG emissions and transportation costs traveled among the waste bins which need to be collected. A selection flowchart is developed to select the waste bins to be collected and determine the collection order based on the priority of waste bins and the data of filling levels of general waste bins from sensors. The flowchart of priority and knowledge-based decision making is presented in Figure 6. It is worth mentioning that sensor-based waste bins are already in use in some developed cities where the proposed model considering priority and filling level is applicable.

In order to verify the PCGVRP model, the case data about waste cited from (Zhang, Ma, Lei and Fu, 2019) are used. There is a disposal center and 30 waste bins with sensors. All vehicles start from the disposal center, and transport all collected waste back to the waste disposal center. The working time window of the vehicle is 19:00–22:00 and during the algorithm running process, its time window is converted into 0 to 180 in minutes. The priority of waste bins is defined randomly. Table 9 gives the detail information of the data including locations, amount of waste and priority of waste bins. The relevant parameters are set as shown in Table 10.

In the following section, five experiments are designed:

- Experiment about different objective functions;
- Experiment about collecting waste in conventional scenario in priority considered scenario;
- Experiment about different number of waste bins with high priority;
- Experiment about different waste filling levels;
- Experiment about different waste filling levels of general waste bins and different number of high priority waste bins.

#### 5.2.2. Experimental Results

- Experiment about Objective Function

**Figure 7:**
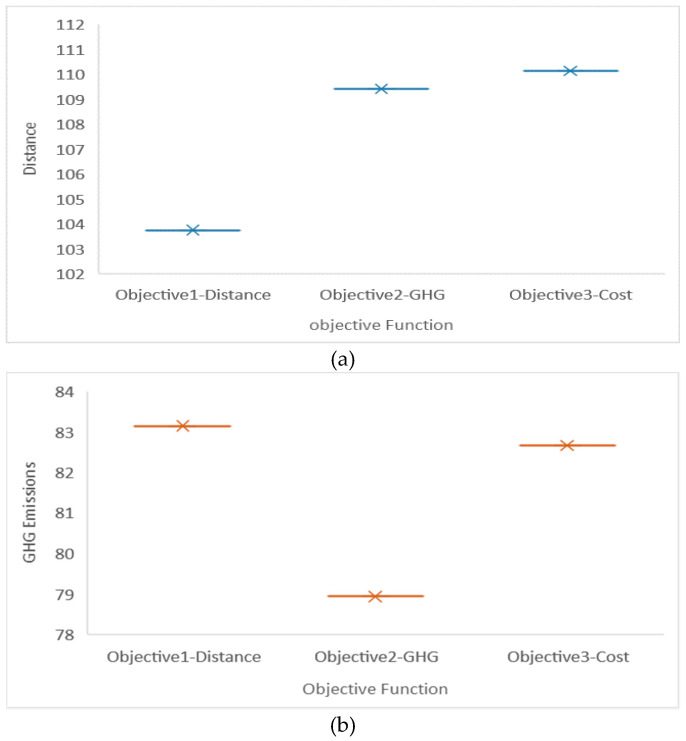

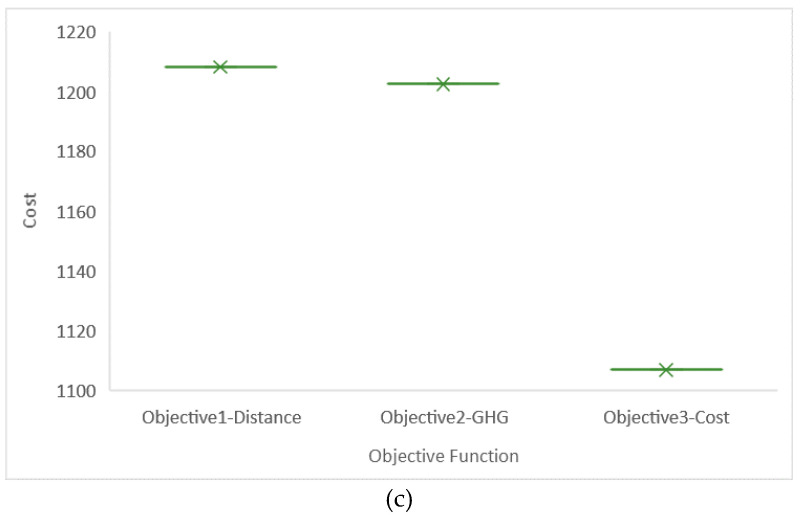
Results comparison with different objectives. (**a**) Objective1-minimized distance, (**b**) Objective2-minimized GHG and (**c**) Objective1-minimized cost.

The first experiment is designed to select objective function: $OF_{1},\;min\;T\_D$; $OF_{2},\;min\;T\_E_{GHG}$; $OF_{3},\;min\;T\_C$. Effects of the different objective functions are shown in Table 11 and the results comparison is illustrated in Figure 7a–c. From these diagrams, we can see that on the one hand, if $OF_{1},\;min\;T\_D$ is selected, distance is shorter than the other two cases while GHG emissions is higher than the one acquired by $TOF_{2},min\;T\_E_{GHG}$. On the other hand, if $OF_{2},\;min\;T\_E_{GHG}$ is selected, the environmental protection level of the routes is optimized, but the distance is longer than the one acquired by $OF_{1},\;min\;T\_D$. For the next experiments, a most common and comprehensive objective function, T_C, will be taken, which means to transform total distance to total fuel cost and transform total GHG emissions cost by a conversion factor.

- Experiment about Conventional Scenario and Priority Considered Scenario

In this section we do the experiment under two scenarios: (1) collect waste in the conventional way (conventional scenario, CS), which means all the waste bins have the same priority without considering the negative effect of specific waste bins; (2) collect waste considering waste bins’ priority (priority considered scenario, PCS). The detailed information about CS and PCS are shown in Table 12 and Table 13, respectively, including service sequence of each route, waste bins with high priority included in each route, the collection order and collection time of the high priority waste bins. From the two tables, we can see that under CS, the order of waste bins with high priority is randomly assigned, resulting in the later collection time. Under PCS, the waste bins with high priority are always first in the collection sequence and then are collected earlier.

**Figure 8:**
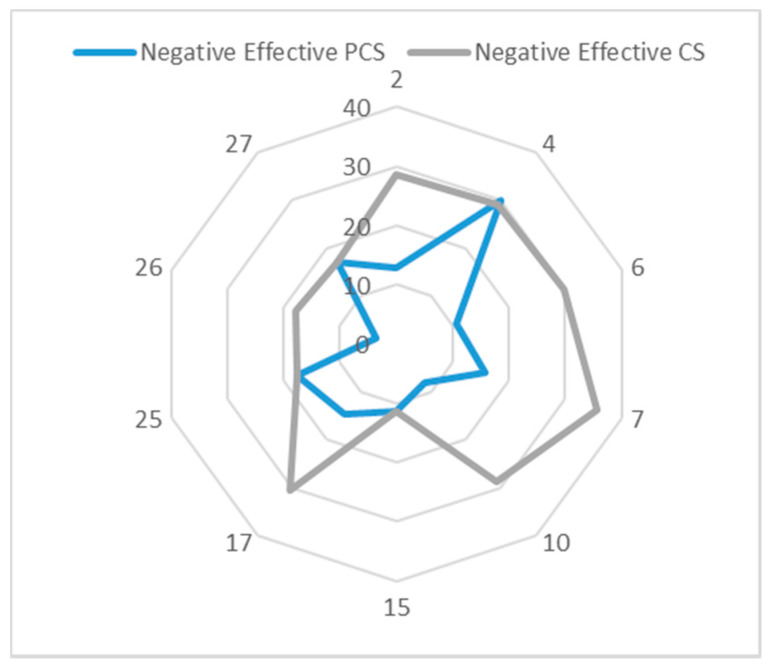
Comparison of negative effect under PCS and CS.

**Figure 9:**
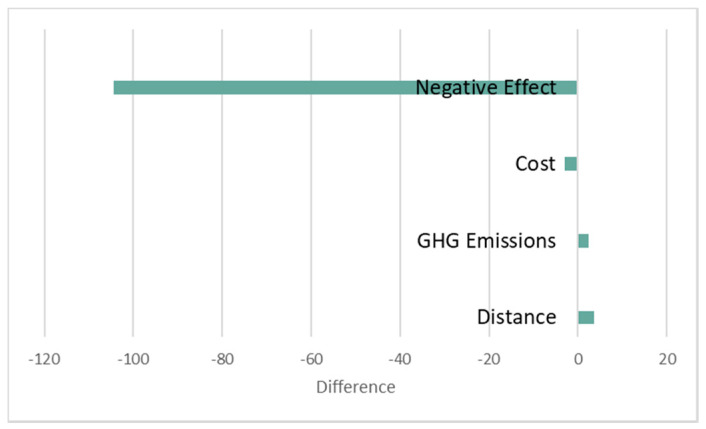
Differences between PCS and CS.

In order to better compare CS and PCS, a new metric is defined. The waste in waste bins with high priority has a negative effective, and the longer time the waste accumulates, the greater the negative effect. Therefore, we quantify the negative effect with the collection time of high priority waste bins. Figure 8 illustrates the effective comparison. We can see that PCSs negative effect is almost surrounded by CSs, which means that almost all the collection time of each high priority waste bin in PCS is earlier than it in CS. In the 10 waste bins with priority, there are six waste bins collected in PCS much earlier than in CS: No.2, No.6, No.7, No.10, No.17, and No.26; there are 3 waste bins with priority collected at the same time both in PCS and CS: No.15, No. 25, and No.27; there is only one waste bin collected in PCS a little later than in CS: No4, which is because the route servicing No.4 waste bin in PCS contains two waste bins with priority while No.4 is at the second place. On the whole, the total negative effect of PCS is 141.72 achieving a 42.3% reduction compared with CS. To prove the overall optimization capability of PCS, besides the comparison of negative effect, Figure 9 gives the difference of distance, GHG emissions, costs and negative effects between PCS and CS. We take this kind of operation with CSs distance, GHG emissions, costs and negative effects MINUS PCSs to obtain the data in Figure 9. We can clearly see that changes in distance, GHG emissions and costs are small enough to be ignored while the negative effect differs greatly. We know the proposed PCS performs better in terms of decreasing negative effect, distance, GHG emissions and costs.

- Experiment about Different Number of High Priority Waste Bins

**Figure 10:**
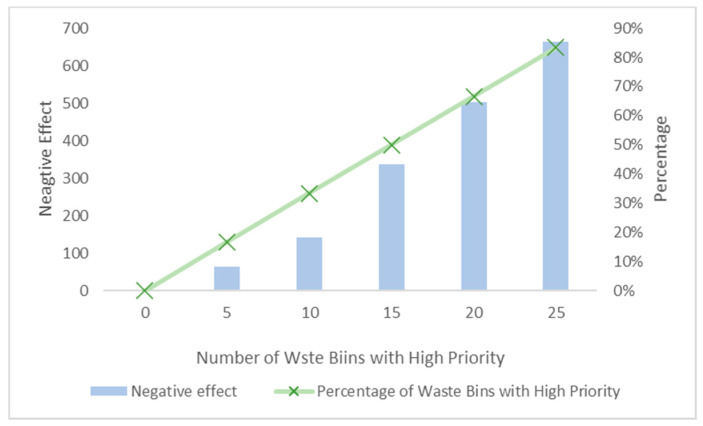
Negative effect under different number of high-priority waste bins.

In order to study the impact of different numbers of high priority waste bins, this section undertakes the experiment considering different numbers including 0, 5, 10, 15, 20 and 25. It is worth mentioning that number of total waste bins is constant, 30. So the percentage of waste bins with high priority increases in Figure 10. We can also see from the figure that the percentage of waste bins with high priority increases as number increases due to the constant total number of waste bins. As the percentage increases, the negative effect increases. This is because that with the number of high priority waste bins increasing, some high-priority waste bins have to share the same car and several of them will be arranged for later collection resulting in the raising of negative effect.

- Experiment about Different Waste Filling Levels (WFLs)

**Figure 11:**
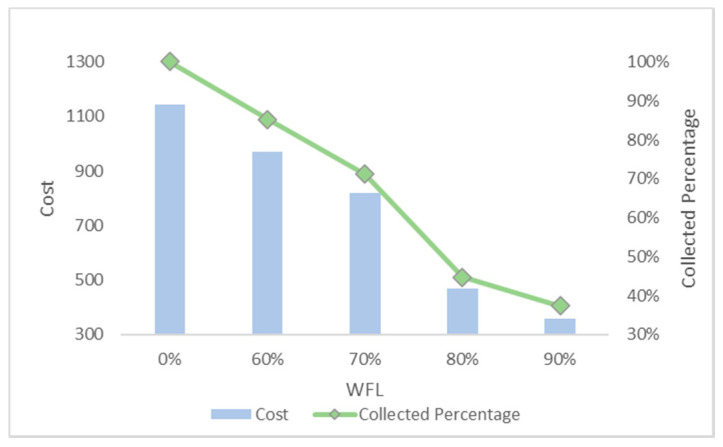
Under different waste filling levels (WFLs).

In this section, we set different values of WFL and the results are shown in Figure 11. Due to the increase of WFL, fewer waste bins reach the threshold and are included in the collection route, resulting in costs and collected waste reduction.

- Experiment about Different WFLs and Different Number of High-Priority Waste Bins

**Figure 12:**
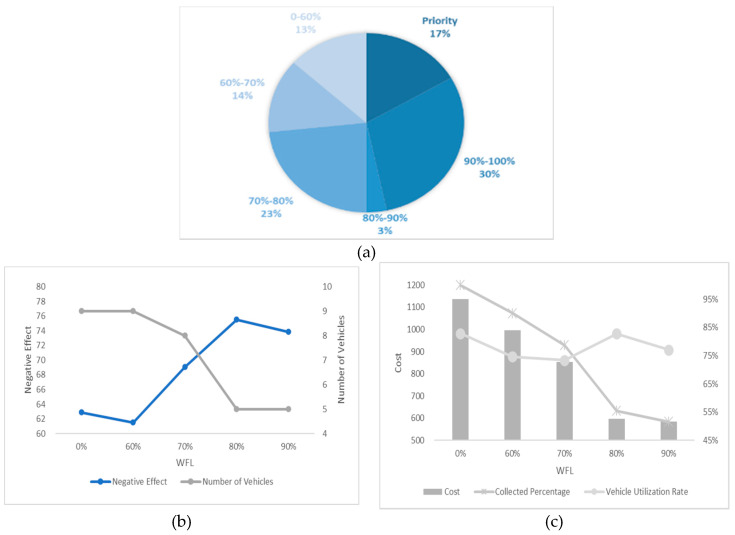
Results with 5 high-priority waste bins. (**a**) PCS-5 distribution of various waste bins, (**b**) PCS-5 negative effect and number of vehicles and (**c**) PCS-5 cost and vehicle utilization rate.

**Figure 13:**
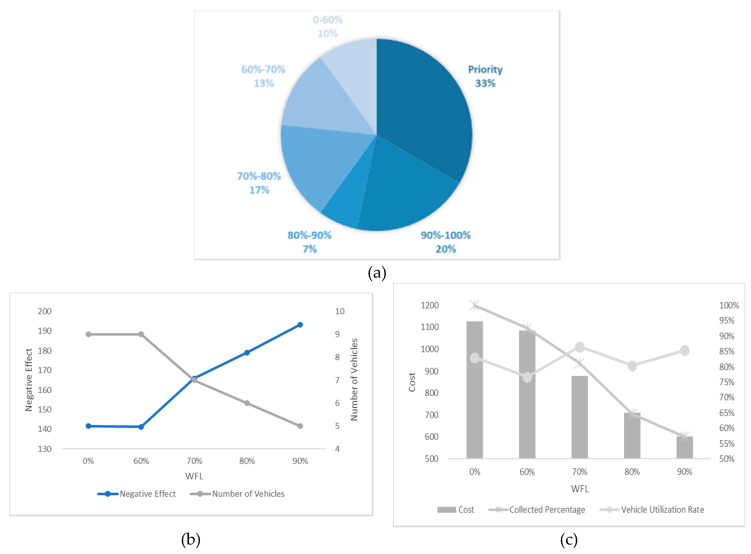
Results with 10 high-priority waste bins. (**a**) PCS-10 distribution of various waste bins, (**b**) PCS-10 negative effect and number of vehicles and (**c**) PCS-10 cost and vehicle utilization rate.

**Figure 14:**
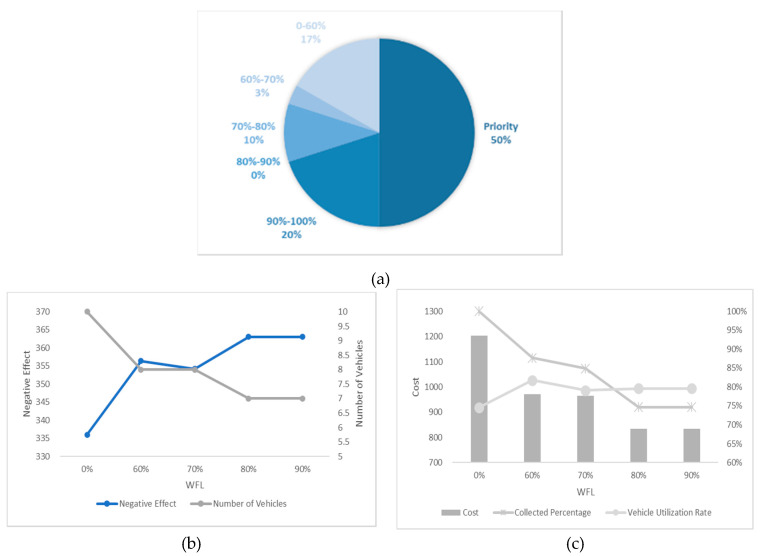
Results with 15 high-priority waste bins. (**a**) PCS-15 distribution of various waste bins, (**b**) PCS-15 negative effect and number of vehicles and (**c**) PCS-15 cost and vehicle utilization rate.

**Figure 15:**
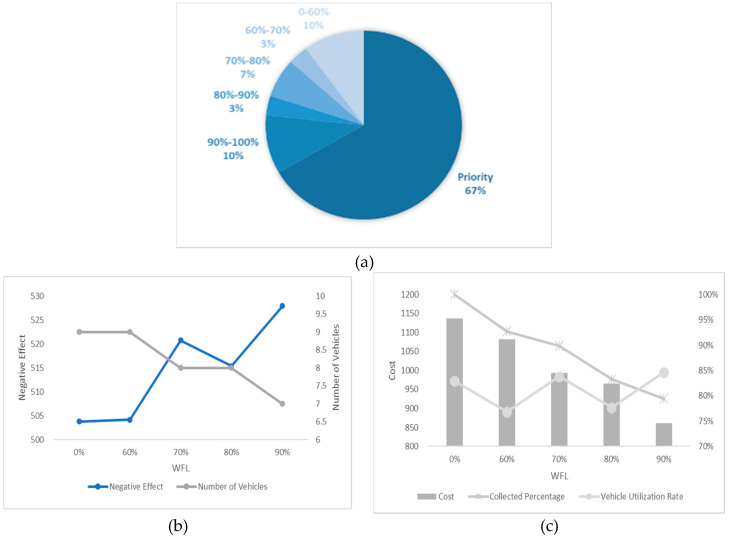
Results with 20 high-priority waste bins. (**a**) PCS-20 distribution of various waste bins, (**b**) PCS-20 negative effect and number of vehicles and (**c**) PCS-20 cost and vehicle utilization rate.

**Figure 16:**
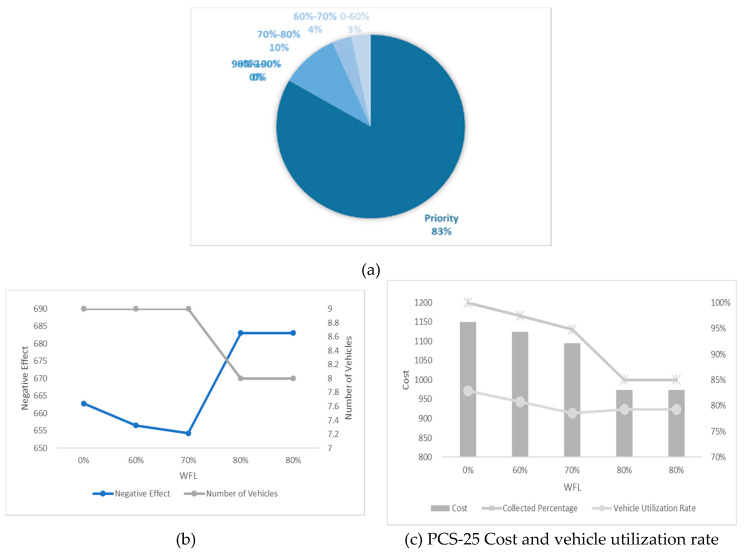
Results with 25 high-priority waste bins. (**a**) PCS-25 distribution of various waste bins, (**b**) PCS-25 negative effect and number of vehicles and (**c**) PCS-25 cost and vehicle utilization rate.

In this section, we undertake an experiment considering different numbers of high-priority waste bins and different WTL at the same time and the results are shown in Figure 12, Figure 13, Figure 14, Figure 15 and Figure 16. It is worth noting that all the waste bins with high priority will be collected no matter what the WFL is. And the leftover waste bins with general priority will operate selective collection according to WFLs. We call each scenario PCS-n. PCS means priority considered scenario and “n” represents the number of waste bins with high priority.

Every number of waste bin with high priority (every figure) has three Figures a–c. For example, a–c in Figure 12 are all sub-Figures of PCS-5. The first sub-Figure of each Figure is a pie chart about the priority and volume distribution. We take Figure 14 as an example and (a) is the first sub-Figure of PCS-15. We can see from Figure 14a that the number of waste bins with high priority accounts for half and the filling level of waste between [0–60%), [60–70%), [70–80%), [80–90%) and [90–100%) accounts for 17%, 3%, 10%, 0% and 20%.

The second sub-Figure of each figure is about the number of vehicles and negative effect and we take (b) of Figure 13 as an example. We set five values of WFL, 0%, 60%, 70%, 80% and 90% as abscissa, which means only the waste bins reaching preset specific WFL will be collected. We study negative effect and number of vehicles changes in the process of increasing WFL. We can see the number of vehicles decreasing as the WFL is increasing as a result of reduction of waste bins to collect. Affected by the decline in the number of vehicles, several waste bins with high priority have to share the same car resulting in the negative effect raising. Therefore, the negative effect and number of vehicles have the opposite trend.

The third sub-Figure is about the cost percentage of collected waste and vehicle utilization rate under different WFL. For the third sub-Figures of all figures, the cost and the percentage of collected waste have the same trend: decreasing as WFL is increasing. That is because when we set higher WFL, fewer waste bin can reach it and will be collected resulting in the lower percentage of collected waste and further reducing cost. Vehicle utilization rate, another indicator, is calculated by dividing total collected waste by total vehicles’ capacity, representing resource utilization efficiency. Different number of waste bins with high priority and WFL lead to different vehicle utilization rate. We can find from all the sub-Figures that there is an effective interval (60–80%) of WFL with high vehicle utilization rate. The logistic enterprises can increase the efficiency of distribution by optimizing the paths when WFL is set in this interval. Further excessive value should be avoided to prevent overflowing before the next collection.

### 5.3. Analysis of Results

In the paper, the PCGVRP model is developed to optimize the activity of waste collection and transportation in SWM, which is solved by the improved algorithm of LSHA. By considering specific waste bins with high priority and filling levels of general waste bins, we study the impact of different numbers of waste bins with high priority and WFLs on the negative effect, costs, the percentage of collected waste and vehicle utilization rate. We find that the best value of WFL with high efficiency should be set according to the number of waste bins with high priority, waste generation rate, waste management requirements and so on. Some primary points of summary are enumerated below.

(1) For experiment 1, the objectives of minimizing distance, minimizing GHG emissions and minimizing costs are compared. The objective of minimizing costs is the compromised one considering both distance and GHG emissions.
(2) For experiment 2, PCS and CS are same in distance reduction, GHG emissions reduction and costs reduction, while PCS achieves a negative effect reduction of 42.3%.
(3) For experiment 3, the negative effect increases as the number of waste bins with high priority is increasing.
(4) For experiment 4, the higher the WFL, the lower the percentage of collected waste and the costs. However, excessive WFL can increase the risk of overflowing before the next collection.
(5) For experiment 5, different distribution of various waste bins (waste bins with high priority, waste filling level of general waste bins) result in different negative effect, costs, vehicles utilization rate and so on. The WFL between 60% and 80% can obtain the optimal solution under different number of waste bins with high priority.

By setting different numbers of waste bins with high priority and different WFLs, it turns out that the proposed PCGVRP model is applicable and efficient for the activity of waste collection and transportation in SWM.

On account of the aforementioned summary, this paper comes up with some instructional advice. From the point of view of SWM institutions, they can take scientific approaches, such as operational research methods, route optimization, etc., to bring down the total negative effect and total costs. At the same time, technical means such as sensors are recommended, because they can bridge the communication between vehicles and waste bins and assist path optimization. In short, waste collection and transportation can greatly benefit from these technologies. A certain value of WFL between 60% and 80% is a preferable option to enhance the efficiency of waste collection and transportation and the exact value of WFL should be decided by the number of waste bins with high priority.

From the perspective of the Environmental Protection Administration, firstly, they should encourage waste management organizations to take the priority of waste bins into consideration and minimize the negative effect of specific waste as much as possible. Secondly, they can introduce some relevant policies to deploy more sensors. Finally, they ought to increase environmental consciousness and inspire the public waste sorting to ensure that priority waste can be sorted, collected and transported in a timely manner.

## 6. Conclusions

Programming a series of vehicle routes well is a challenging task in an effort to decrease collection and transportation costs, the negative effects of some specific waste and to ensure that all inhabitants live in a comfortable and healthy environment. Thus, the paper built a model for waste collection and transportation with the minimized total comprehensive costs including routes costs and GHG emissions costs. An improved genetic algorithm, LSHA, is designed to solve the proposed PCGVRP model. Furthermore, the effectivity of LSHA is proved by a contrast experiment using data from classic CVRP database. After that, the applicability and validity of the proposed PCGVRP model are confirmed by setting different numerical parameters. The negative effect, costs, vehicles utilization rate and percentage of collected waste are computed and analyzed separately with different numbers of waste bins with high priority and WFLs. Bases on the results, some suggestions are provided for the Environmental Protection Administration and waste collection and transportation organizations. In the case of COVID-19, the increase of medical waste makes the timely disposal of this waste particularly important and critical. In further research, multivariate statistical analysis (cluster analysis and principal component analysis) can be applied to analyze the parameters of PCGVRP Furthermore, recent and real data can be used to obtain more realistic and reliable conclusions.

## Figures and Tables

**Table 1:** Notation of the priority considered green vehicle routing problem (PCGVRP) model.

Sets	Unit	Description
B		Set of waste bins ($B=b_{1},\;b_{2},\cdots ,\;b_{i},\cdots ,b_{n}$)
D		Waste disposal center
V		Set of vehicles ($V=v_{1},\;v_{2},\cdots ,\;v_{k},\cdots ,v_{K}$)
$C_{p}$	kg	Maximum load capacity of vehicle
T_D	km	Total distance of all vehicles
$T\_E_{GHG}$	kg	Total GHG emissions of all vehicles
T_C	CNY	Total cost of all vehicles
ϵ	CNY/kg	Cost of greenhouse gas (GHG) emissions per unit
e	$\mathrm{kg}\;CO_{2}e/L$	Emission coefficient
r	L/km	Fuel consumption rate per unit distance
$r_{0}$	L/km	Fuel consumption rate per unit distance while vehicle is empty
$r^{*}$	L/km	Fuel consumption rate per unit distance while vehicle is at full load
r(Q)	L/km	Fuel consumption rate per unit distance with load of Q
$r_{ij}$	L/km	Fuel consumption rate per unit distance while vehicle goes from waste bin i to j
$E_{GHG}$	kg	GHG emissions
$q_{j}$	kg	Collected waste at waste bin j
$d_{ij}$	km	Distance between waste bin i and j
$t_{i}^{k}$	s	Time of vehicle k arriving at waste bin i
$\lambda_{i}$		If waste bin i has a high priority, $\lambda_{i}=1$. Otherwise, $\lambda_{i}=0$
$P_{fixed}$	CNY	Fixed cost of each vehicle
$P_{fuel}$	CNY/kg	Price of fuel consumption
Variable		
$x_{ij}^{k}$		Whether a vehicle k goes from waste bin i to j

**Table 2:** Particle coding.

Waste Bin Number	1	2	3	4	5	6	7	8	9	10
$X_{a}$	1	1	1	2	2	2	2	3	3	3
$X_{b}$	1	3	2	3	1	2	4	2	3	1

**Table 3:** Particle decoding.

Vehicle Number	Vehicle Route
1	0-1-3-2-0
2	0-5-6-4-7-0
3	0-10-8-9-0

**Table 4:** Parameters of particle swarm optimization (PSO).

Parameters of PSO	Description
$it_{max}$	Maximum number of iterations
lPar	Length of particle code
nPop	Number of population
$r_{1}$	Learning factor1
$r_{1}$	Learning factor2
$c_{1}$	Acceleration factor 1
$c_{1}$	Acceleration factor 2
$v_{max}$	Maximum velocity
$v_{min}$	Minimum velocity
$\left[x_{min},x_{max}\right]$	Particle range
ω	Inertia weight
$r_{\omega }$	Inertia weight damping ratio

**Table 5:** Data about the test instances.

Problems	Case	Node	Capacity
Pro 1	E-n22-k4	22	6000
Pro 2	E-n23-k3	23	4500
Pro 3	E-n30-k4	30	4500
Pro 4	E-n33-k4	33	8000
Pro 5	E-n51-k5	51	160
Pro 6	E-n76-k8	76	180
Pro 7	E-n76-k10	76	140
Pro 8	E-n101-k8	101	100

**Table 6:** Parameters of vehicles.

Parameter	Value
e	3.15 $\mathrm{kg}\;CO_{2}e/L$
$r_{0}$	0.16 L/km
$r^{*}$	0.377 L/km
ϵ	0.025 CNY/kg
$P_{fixed}$	100 CNY
$P_{fuel}$	8 CNY/kg

**Table 7:** Parameters of LSHA.

Parameter	Value
$it_{max}$	1000
nPop	20
$c_{1}$	1.5
$c_{1}$	1.5
$r_{\omega }$	0.99
$T_{0}$	200
α	0.98
$T_{end}$	1
M	5

**Table 8:** Test results of PSO and LSHA.

Problems	PSO			LSHA		
	Distance	GHG Emissions	Cost	Distance	GHG Emissions	Cost
Pro 1	649.40	521.70	1738.01	603.72	467.87	1589.71
Pro 2	946.06	737.81	2192.26	900.32	618.46	1886.17
Pro 3	1161.74	884.11	2567.48	1091.85	783.08	2308.36
Pro 4	1352.04	1028.88	3038.76	1210.99	971.73	2892.19
Pro 5	1491.31	1211.97	3608.33	1383.12	1148.54	3402.13
Pro 6	2331.47	1841.55	5622.99	2115.12	1746.72	4936.27
Pro 7	2298.04	1746.65	5779.60	2089.77	1532.66	5043.73
Pro 8	3152.75	2636.75	7562.42	2836.39	2339.05	6411.86

**Table 9:** Information about the disposal center and waste bins.

Point	X Coordinate	Y Coordinate	Amount of Waste (kg)	Priority
Disposal Center	4.8	4.74	—	—
1	0.98	0.08	626.87	General
2	3.6	1.05	566.31	High
3	3.35	2.68	772.5	General
4	1.92	4.27	913.9	High
5	2.46	4.55	918.5	General
6	3.87	1.67	916.67	High
7	0.74	2.35	601.86	High
8	2.43	0.01	772.21	General
9	0.36	1.55	937.47	General
10	3.94	2.43	560.5	High
11	1.97	1.31	928.18	General
12	1.18	3.42	949.89	General
13	0.4	4.56	608.93	General
14	0.4	2.85	538.49	General
15	4.64	1.33	737.11	High
16	1.02	4.64	917.51	General
17	4.78	0.32	734.7	High
18	1.7	3.13	706.88	General
19	0.3	0.54	751.37	General
20	1.72	2.58	562.72	General
21	3.02	4.78	566.14	General
22	3.06	1.36	935.24	General
23	2.78	2.63	801.48	General
24	2.73	3.56	632.65	General
25	1.01	1.07	932.4	High
26	3.94	4.13	529.05	High
27	1.74	0.69	728.88	High
28	4.86	2.19	861.1	General
29	0.58	3.88	669.5	General
30	3.56	3.52	700.61	General

**Table 10:** Parameter setting.

Parameter	Value
t	5 min
$P_{fixed}$	100 CNY
v	30 km/h
Capacity	3000 kg

**Table 11:** Running results with different objectives.

	Objective1-Distance	Objective2-GHG	Objective3-Cost
Distance	**103.7554**	109.4249	110.1329
GHG Emissions	83.1519	**78.9363**	82.6705
Cost	1208.129	1202.4465	**1106.894**

Highlighted values represent the optimal values of distance, GHG emissions and cost with different objectives.

**Table 12:** Detailed route information under conventional scenario (CS).

Number	Routes	High Priority Waste Bin	Collection Order	Collection Time
1	{6,24,9,021,24,9,0}	17	2nd	30.54
2	{0,**15**,11,7,0}	15	1st	11.38
		7	3rd	35.65
3	{0,8,**4**,12,0}	4	2st	29.03
4	{0,**25**,14,0}	25	1st	17.59
5	{0,13,29,22,0}	-	-	-
6	{0,**27,6**,28,0}	27	1st	16.92
		6	2nd	29.74
7	{0,23,16,30,0}	-	-	-
8	{0,5,**26**,**10**,0}	26	2nd	17.95
		10	3rd	28.62
9	{0,3,**2**,0}	2	2nd	28.62
10	{0,20,19,1,0}	-	-	-
Total				246.03

**Table 13:** Detailed route information under priority considered scenario (PCS).

Number	Routes	High Priority Waste Bin	Collection Order	Collection Time
1	{0,**27**,5,11,0}.	27	1st	16.92
2	{0,**15**,1,30,16,0}	15	1st	11.38
3	{0,**10**,8,20,12,0}	10	1st	8.22
4	{0,**7**,29,14,24,0}	7	1st	15.70
5	{0,**25**,28,3,0}	25	1st	17.59
6	{0,**17**,22,19,23,0}	17	1st	14.73
7	{0,**2**,**4**,21,9,0}	2	1st	12.93
8		4	2nd	30.04
9	{0,**26**,18,13,0}	26	1st	3.52
10	{0,**6**,0}	6	1st	10.69
Total				141.72
